# The 7 Elements Communication Rating Form for Teaching and Assessing Medical Communication Competency

**DOI:** 10.7759/cureus.59008

**Published:** 2024-04-25

**Authors:** Christina M Wise, Gretchen D Lovett, Gail M Swarm, Andrea Nazar

**Affiliations:** 1 Department of Clinical Sciences, West Virginia School of Osteopathic Medicine, Lewisburg, USA

**Keywords:** communication in healthcare, communication assessment tool, interpersonal and communication skills, medical communication checklist, humanistic domain, osce, communication skills assessment, teaching communication skills

## Abstract

Introduction: Medical communication skills are a critical component of clinical medicine and patient satisfaction. Communication skills are difficult to teach and evaluate, necessitating tools that are effective and efficient. This study presents and validates the 7 Elements Communication Rating Form (7E-CRF), a streamlined, dual-purpose, evidence-based medical communication checklist that functions as a teaching and assessment tool.

Method: A 14-item teaching and assessment tool is described and validated using face, concurrent, and predictive validity indices. The study was conducted with 661 medical students from the West Virginia School of Osteopathic Medicine (WVSOM). Student performance was assessed in year 1 labs, year 2 labs, and year 2 and year 3 objective structured clinical examination (OSCE). These internal indices were compared with student performance on the Humanistic Domain of the Comprehensive Osteopathic Medical Licensing Examination (COMLEX) Level 2-Performance Evaluation (PE), a licensure exam previously taken in years 3 or 4 of osteopathic medical schools.

Results: The evidence of interrater reliability and predictive validity is strong. Data from the 7E-CRF is compared to performance on the COMLEX Level 2-PE, Humanistic Domain. The 7E-CRF can identify students who are at a 10-fold increased risk of failure on the COMLEX Level 2-PE Humanistic Domain.

Conclusions: The 7E-CRF integrates instruction and assessment, based on a national and international model. The simplicity, foundation in professional consensus, ease of use, and predictive efficacy make the 7E-CRF a highly valuable instrument for medical schools in teaching and evaluating competency in medical communication skills.

## Introduction

There has been increased attention to medical communication skills in medical education stemming from the core competency movement promulgated by the Accreditation Council of Graduate Medical Education [1]. Gaining momentum since the turn of the millennium, this important area has been in need of tools for assessing medical communication skills as well as a concise training model for novice learners in medicine and advanced practitioners alike [2]. Over 70 published tools exist to assess medical communication skills with varying theoretical bases, design strategies, and reliability data [3,4].

Bedside manner has been a construct in the discussion of doctoring throughout the 20th century and into the new millennium [5,6]. Many people have family members who have been treated by doctors who they regard as having wonderful bedside manners and others they find lacking in this critical domain. Indeed, patient satisfaction surveys report that the doctor’s bedside manner is a critical factor in choosing a practitioner [7]. Prior to 2000, there was substantial heterogeneity in training on healthcare communication strategies with different schools teaching different concepts, and with different professors within a particular medical school teaching different approaches. Such variety and variability did not lend itself to standardization and competency-based approaches that were growing in medical education [8].

The culture of academic medicine has increasingly elevated doctor communication skills as part of its purview [9]. Standardized curricula for teaching medical communication skills and tools for measuring such skills are essential, particularly as communication competency and the evaluation of communication skills has been a requirement for medical licensure in both the Osteopathic and Allopathic professions [10]. Although both Doctor of Medicine (MD) and Doctor of Osteopathic Medicine (DO) licensure organizations have pulled back from clinical examinations (partly due to COVID-19 pandemic considerations), both licensing organizations (NBOME and NBME) continue to work with medical schools to standardize and ensure communication competency as part of their charge to protect the public.

The increased importance of communication competency and high-stakes examinations made standardization across medical schools critical. In 2001, The Kalamazoo Consensus Statement, by the participants in the Bayer-Fetzer Conference on Physician-Patient Communication in Medical Education, brought about a desperately needed professional agreement that detailed the “essential elements of healthcare communication” [11]. These seven elements are 1) open the discussion, 2) build the relationship, 3) gather information, 4) understand the patient’s perspective, 5) share information, 6) reach agreement on problems and plans, and 7) provide closure. These elements also form the backbone of the didactic material on DocCom, a widely used internet platform for teaching medical communication skills produced by the Academy on Communication in Healthcare [11,12].

The objective of this study is to describe and validate an effective rating score for accessing clinical communication skills of osteopathic medical students and residents. An instrument was designed with the goals of reliability, validity, brevity, clarity, and facility of training and use.

## Materials and methods

Setting

The study was conducted with 661 medical students from the West Virginia School of Osteopathic Medicine (WVSOM), Lewisburg, United States in the classes of 2016, 2017, and 2018. The medical students were educated, trained, and practiced on the 7 Elements Communication Rating form (7E-CRF) from the beginning of their first year. The 7E-CRF was assessed in year 1 formative labs, year 2 formative labs, and in summative assessments in the year 2 and year 3 objective structured clinical examinations (OSCE). WVSOM Institutional Review Board issued approval (2018-3).

Design of the 7E-CRF

In light of the elegant framework provided by both the Kalamazoo Consensus Statement and DocCom, WVSOM chose the structure of the essential elements for its didactic and evaluative communication tool development [11,12]. The seven essential elements formed the structure of the assessment tool and training model. On the 7E-CRF, each essential element is a domain with two specifically designed items creating a 14-item scale. For the design of the items, the committee had three considerations (1) face validity and consistency of the item with the description provided in the Kalamazoo Consensus statement, (2) research evidence of the item, and (3) ease of training for both raters (standardized patient (SP), faculty, students and administrators) and learners. 

The inclusion of two scored items for each of the elements resulted in an evaluation tool with 14 items for grading in seven domains (Table 7). Among the 14 items, some are more “objective” while others are more “subjective.” The objective in this sense means that they are more clearly anchored in discrete, observable behaviors (e.g., did the student introduce themselves by name). The more subjective items, on the other hand, are multi-faceted, nuanced, and essentially a Likert Scale with the categories of poor (1), fair (2), good (3), and excellent (4). These more subjective items tend to be more global in nature, have a higher level of consideration grading, and require the SP to make a judgment based on their experience and expertise in grading tens to hundreds of students, thus comparing a new student to an internalized sense of the distribution of performance. The SPs receive orientation training on the form, written grading guidelines, and continued monthly training on the communication rating form in addition to the specific case training, following Aspe’s best standards of practice [13].

For each of the 14 items on the scale, the grader must select a response of 1, 2, 3, or 4. These are not all associated with consistent qualitative words since, as described above, some of the items fan out across a qualitative descriptive gradation, while others relate to the presence or absence of identified target behaviors in the sample of student behavior. These point values are then recalibrated to a 10-point scale for evaluative and grading purposes with 1 = 4, 2 = 6, 3 = 8, and 4 = 10. With this calibration, we are able to render scores on a 10 or 100-point distribution which works well with WVSOM grading practices with a score of 70% to 75% generally used for passage of various high-stakes assessments. The 14-item 7E-CRF translates the scores to a scaled score with 10 as perfect performance per item which provides for a maximum score of 140 on the overall instrument. This is converted to a 100-point or percentage score which corresponds well to assessment in the WVSOM curriculum.

Use of the 7E-CRF for teaching and evaluation

Students receive instruction on the 7E-CRF beginning the first week of their first year in medical school and have continuous access to the form. The decision was made to disregard the students’ educational level when assigning scores on the 7E-CRF and use the 7E-CRF as a consistent measurement tool. The rationale for this decision is two-fold. First, the reliability of ratings on a measure of such complex, interactive human behavior is always a challenge and requires intensive, continuous training. Asking raters (primarily SPs) to alter their scoring judgments based on a student’s year in the curriculum would add an unnecessary challenge and unreliability to the scoring activity. This is compounded with the fact that the 7E-CRF is also used for residents and practitioners; so differentially adapting a unique version of the scoring judgments for each group seemed a cumbersome invitation to "police" and reduced reliability. Also, the concept of a consistent measurement tool allows student progress over the course of the curriculum to be followed. In this model, a student, and the institution, can see how a student’s skills change over the course of their education and clinical exposure, and also in relation to experimental educational interventions and curricular changes.

The intensive training and reliability process for the SPs has been largely conducted as a live, group activity with a detailed manual for guidance. The manual has scoring guidelines and examples for each of the 14 items as well as philosophical orientation and justifications of the approach. The manual provides the SPs with insight into how the students are trained in communication. Knowing the information the student is given beforehand reassures the SP that the student is being graded fairly and encourages them to grade accurately. The information in the manual is crucial for the consistency of graders [14].

The reliability of the 7E-CRF is of ongoing importance. Continual training is essential to maintain the interrater reliability of a group of 60 to 80 SPs. A “Reliability Workshop” is held monthly where SPs come together for three hours of training with dedicated faculty and simulation center staff. The Reliability Workshop is a costly endeavor but necessary to maintain interrater reliability.

During the workshop each month, one case from our year 3 OSCE is reviewed. Three videos of this case are observed and all the present SPs grade the student using the 7E-CRF. After grading is complete, each item is verbally reviewed and the SPs are able to visually see if they are with the majority or could possibly be grading too hard or too easy. The discussion gives the SPs the ability to understand what is important for each question and how others may have interpreted the same actions. The data gathered at each Reliability Workshop is collected and used to evaluate the SPs individually and as a group.

## Results

7E-CRF performance

Overall average scores on the 7E-CRF have been analyzed by curricular year for the class of 2016, 2017, and 2018. Table 1 shows the average 7E-CRF scores for an eight-station OSCE for year 2 and a 12-station OSCE for year 3.

**Table 1:** Average 7E-CRF score by graduating class for year 2 and year 3 OSCE The average scores for a second-year eight-station OSCE and a third-year 12-station OSCE for academic years 2016, 2017, and 2018. OSCE: objective structured clinical examination; 7E-CRF: 7 Elements Communication Rating Form

Class	Year 2 OSCE	Year 3 OSCE
2016	78.3%	80.3%
2017	79.0%	82.3%
2018	80.6%	80.3%

On average, students are scoring between 7.8 and 8.0 on each item, which corresponds to the Likert designation of just under a “3.” For the class of 2016 and 2017, there appears to be a slight trend toward improved 7E-CRF performance from year 2 to year 3 OSCE, but this was not found in the class of 2018. No significant effect was found for the gender of students with male and female averages for the class of 2017 during the year 3 OSCE being very similar (female = 82.6; male = 82.0). 

7E-CRF predictability of pass vs fail on Comprehensive Osteopathic Medical Licensing Examination (COMLEX) Level 2-Performance Evaluation (PE)

The predictive validity of the 7E-CRF was analyzed by comparing WVSOM year 3 OSCE scores on the 7E-CRF to student performance on the National Board of Osteopathic Medical Examiners COMLEX Level 2-PE, Humanistic Domain. WVSOM has consistently performed well on the COMLEX Level 2-PE Humanistic Domain with pass rates in the 97-98% range (class of 2016 = 97.5%; class of 2017 = 97%; class of 2018 = 98%). With such a large percentage of the student body passing this critical exam, we have looked closely at those who do not pass and considered their 7E-CRF scores to determine if predictions could be made. Among the class of 2017 (sample size 177), there was a statistically significant (p < 0.001) difference between 7E-CRF scores for the year 3 OSCE students who passed the Humanistic Domain (82.6) and those who failed the Humanistic Domain (75.9) of the COMLEX Level 2-PE.

A useful method for analyzing the data is to convert 7E-CRF scores via a cutoff score of 75 into categorical variables such as high and low performers. Using this dichotomy and comparing the high and low performers with COMLEX 2-PE pass/fail yields a 2 × 2 matrix of hits and misses. In the class of 2018 year 3 OSCE, 15 out of 198 students scored <75 on the 7E-CRF for year 3 OSCE. Two of the 15 failed the PE Humanistic Domain and 13 did not. Additionally, two students who scored above 75 on the 7E-CRF failed the PE Humanistic Domain. This data can be analyzed in terms of “hits” and “misses.” (a) Low performers on the 7E-CRF had a 2 in 15 or 13% chance of failing the Humanistic. (b) High performers on the 7E-CRF had a 2 in 183 or 1.1% chance of failing the Humanistic. (c) Therefore, low performance on the 7E-CRF is associated with an approximately 11.8-fold increased risk of failing the PE Humanistic Domain.

This similar analysis is presented in Tables 2, 3, 4 for each of the graduating classes of 2016, 2017, and 2018.

**Table 2:** Humanistic Domain result by 7E-CRF status-class of 2016 (n = 183) The class of 2016 comparison results with 7E-CRF score and performance on COMLEX PE including “hits” and “misses.” 7E-CRF: 7 Elements Communication Rating Form; PE: Performance Evaluation

7E-CRF performance	COMLEX PE humanistic pass	COMLEX PE humanistic fail	COMLEX PE humanistic pass or fail
High performers	165 true negatives	3 misses	168
Low performers	13 false positives	2 hits	15
All CRF scores	178	5	183

**Table 3:** Humanistic Domain result by 7E-CRF status-class of 2017 (n = 180) The class of 2017 comparison results with 7E-CRF score and performance on COMLEX PE including “hits” and “misses.” 7E-CRF: 7 Elements Communication Rating Form; PE: Performance Evaluation

7E-CRF performance	COMLEX PE humanistic pass	COMLEX PE humanistic fail	COMLEX PE humanistic pass or fail
High performers	170 true negatives	3 misses	173
Low performers	4 false positives	3 hits	7
All CRF scores	174	6	180

**Table 4:** Humanistic Domain result by 7E-CRF status-class of 2018 (n = 198) The class of 2018 comparison results with 7E-CRF score and performance on COMLEX PE including “hits” and “misses.” 7E-CRF: 7 Elements Communication Rating Form; PE: Performance Evaluation

7E-CRF performance	COMLEX PE humanistic pass	COMLEX PE humanistic fail	COMLEX PE humanistic pass or fail
High performance	181 true negatives	2 misses	183
Low performance	13 false positives	2 hits	15
All CRF scores	194	4	198

The increased risk of failing the COMLEX Level 2-PE Humanistic Domain is substantial for individuals who score at or below an overall year 3 OSCE 7E-CRF score of 75%. The increased risk for each of these classes is shown in Table 5.

**Table 5:** Increased risk of failing the COMLEX Level 2-PE Humanistic Domain as a function of a low 7E-CRF score The increased risk of failing the humanistic domain is substantially increased for students scoring at or below 75% as shown in the table. 7E-CRF: 7 Elements Communication Rating Form; PE: Performance Evaluation

Risk of failing the COMLEX Level 2-PE Humanistic Domain			
Class	Above 75 7E-CRF	Below 75 7E-CRF	Increased Risk for students below 75 7E-CRF
2016	1.8%	13%	7.2-fold (720%)
2017	1.7%	43%	25.3-fold (2530%)
2018	1.1%	13%	11.8-fold (1180%)

The 7E-CRF status of high versus low performance was predictive of risk for COMLEX 2-PE failure with lower performance being 7 to 25 times more likely to fail as compared to 7E-CRF high performers. 

7E-CRF reliability analysis 

Data from SP Reliability Workshop monthly coding of year 3 OSCE encounters in 2016 (class of 2017) indicate interrater reliability range from Cronbach’s Alpha of 0.69 to 0.83 on individual third-year OSCE stations [15]. As seen in Table 6, in 2017, year 3 OSCE stations’ 7E-CRF reliability values were mostly above 0.70 value. Overall internal reliability for the 7E-CRF is calculated at a Cronbach's Alpha value of 0.75.

**Table 6:** Interrater reliability by OSCE station Year 3 OSCE stations for the class of 2017 indicate an interrater reliability range of Cronbach's Alpha of 0.69 to 0.83 with an overall value of 0.75. OSCE: objective structured clinical examination

Station	Cronbach’s Alpha
1	0.76
2	0.75
3	0.83
4	0.79
5	0.64
6	0.80
7	0.76
8	0.71
9	0.69

## Discussion

In this study, a tool for teaching and assessing healthcare communication is described [16]. The use of the same tool for both teaching and evaluation is a critical component of the 7E-CRF’s success. This “dual-use” provides a continuous loop between instruction and evaluation that is well-suited for adult learners. The reliability of the 7E-CRF is good, and it is also valid with significant predictive validity for identifying at-risk status for the Humanistic Domain of the COMLEX Level 2-PE.

Comparing the 7E-CRF to other instruments, the fundamental advantages are its simplicity (both the teaching model and the evaluation are the same two-page document), the dual-use function (which closes the loop for adult learners), the existence of a detailed SP user manual and proven efficacy in identifying at-risk individuals. An additional aspect upon comparison is the osteopathic philosophy of treating the whole person, whereas each individual is a unified combination of mind, body, and spirit [17]. To fully engage at the “whole person” level, practitioners need effective communication instruction, feedback, and evaluation; the 7E-CRF provides this.

Students who score at or below an overall score of 75 on the 7E-CRF are required to attend additional training in medical communication skills prior to taking the COMLEX Level 2-PE. This re-education program provides additional training and practice with the 7E-CRF model. Without this additional training, many more students with low 7E-CRF scores may have had difficulty at the COMLEX Level 2-PE. It is our hypothesis that this intervention program for these at-risk students contributed to our “false positive” numbers in the analysis in Table 4. Furthermore, in this application, false positives carry little negative consequence viz two hours of extra communication training; for this reason, minimizing false positives (at the expense of fewer hits) is not recommended.

One of the goals of the 7E-CRF is that the tool stands as a highly condensed pedagogical summary of a nationally acknowledged communication model. It is presented repeatedly in student training in a simplified, streamlined, and repetitive spiraling curriculum allowing students to clearly understand and exhibit the communication behaviors they will be tested on. It is a teaching tool in addition to an assessment measure. The students’ attention is frequently called to the fact that this content has been condensed down to a two-page format and that it has a very high yield in terms of reliably predicting board passage while also being brief and easy to use by multiple assessors (students, faculty, and SPs).

The recent change in the elimination of the practical examination component of the COMLEX licensing exam will require medical schools to ensure student competency in clinical skills. The 7E-CRF is demonstrated to be a valid evaluation tool with established cut-off scores that correspond closely to the previous vigor of the COMLEX 2-PE Humanistic Domain standards. Continued studies will need to be completed to further revise the 7E-CRF to accommodate the telehealth platform and additional studies on the competency needed for those encounters.

## Conclusions

The 7E-CRF accomplishes the intended goals of transparency for adult learners, based on the internationally accepted Kalamazoo Model for best practice, ease of use, and strong reliability and validity. Thus, it is a prime tool for adoption across educational and healthcare settings.

## Appendices

**Table 7:** 7 Elements: WVSOM Communication Rating Form WVSOM: West Virginia School of Osteopathic Medicine

	7 Elements: WVSOM Communication Rating Form
	Under each item number, there are descriptions of each score level, 1 being the lowest and 4 being the highest. These are given as a general anchor point. Many times, the situation will not fit perfectly into one of these 4 ratings. Also, the grader has the flexibility to raise or lower a grade around this "target example". For instance, if the student meets the stated criteria for a score of 3 on Item #9 (Gives and explains a diagnosis/condition/symptoms/etc.), but uses a lot of jargon, then the grader could bump that grade down from a 3 to a 2.)
Open the Discussion	
1	Greet Patient (greet patient by name, introduce self, eye contact/smile, and sit down) these 4 things must be done prior to eliciting the chief complaint.
	1- Performs 2 or fewer prior to eliciting chief complaint.
	2- Performs 3/4 required elements prior to eliciting chief complaint.
	3- Performs 4/4 required elements prior to eliciting chief complaint.
	4- Performs 4/4 required elements prior to eliciting chief complaint and shows very high sincerity and engagement.
2	Elicit chief complaint and any other concerns using open-ended questions and empathy. "What brings you in today?" " I'm sorry to hear that." "Can you tell me more about that?" "Is there anything else you would like to discuss today?"
	1- Begins with 1 open-ended question.
	2- Begins with 2 open-ended questions.
	3- Asks 3 open-ended questions before the physical exam.
	4- Asks 3 open ended-questions before physical exam and makes an empathy statement.
Build the Relationship	
3	Demonstrate nonverbal warmth. (eye contact, facial expressions, volume/tone of voice, posture/distance, pace, draping, assisting, sitting, appropriate use of Electronic Health Record-EHR, etc.).
	1- Poor nonverbal behavior.
	2- Fair nonverbal behavior.
	3- Good nonverbal behavior.
	4- Excellent nonverbal behavior.
4	Demonstrate verbal warmth through relationship building comments (attentiveness, empathy, respect, support, legitimation, partnership). "I'm sorry to hear that." "I'm sure that must be difficult." "That is a good question." "I will be here to help you through this." "We will see what we can do to get you feeling better today." "We will work together."
	1- No use of relationship building comments.
	2- Minimal use of relationship building comments.
	3- Good use of relationship building comments.
	4- Excellent (frequent and thoughtful) use of relationship building comments.
Gather Information	
5	Ask questions clearly, directly, and one at a time. (avoid leading questions, avoid double questions).
	1- Frequent use of leading or double questions (twice or more).
	2- Infrequent use of leading or double questions (once).
	3- Asks questions clearly and one at a time (no double or leading questions).
	4- Above plus: Covers appropriate social content (typically at least habits, occupation, relationships, safety).
6	Show an organized approach and use transitional statements. (examples: "Ok, now I would like to ask you about your past medical history." "Ok, I'd like to ask you some personal questions just to get to know you a bit better if that’s alright.” "Ok, let's go ahead and start the physical examination if that's OK with you.") Note: it is fine to ask historical or associated symptom questions during the physical.
	1- Sections unclear with no use of transitional statements.
	2- Some sections with no or little use of transitional statements.
	3- Basic sections with at least 1 transitional statement.
	4- Clear sections with 2 or more transitional statements.
Understand the Patient's Perspective (note: this can occur during Hx gathering or after PE)	
7	Discuss the patient's perspective of the illness. (concerns, beliefs, agenda, impact, fear, anger, sorrow, joy) E.g. "How have you been doing with all of this?" "What worries you most?" "What is your biggest concern?" "What do you think could be causing this?"
	1- No mention of patient perspective about the situation
	2- Some acknowledgement of patient perspective (e.g. "You seem pretty worried about this.")
	3- Asks about patient perspective (e.g. "How has it been for you coping with all of this?")
	4- Helps patient process reaction to the illness (active listening, clarifying, legitimizing)
8	Discuss the practical aspects of the illness. (e.g. Home, Work, Driving) e.g. "How has this illness affected your life at home?" "How has this illness affected your work?" "Have you been able to go to work?" "Is missing work affecting you financially?" Need a Doctors note?" "Do you have stairs at home?" "Is there someone who can help take care of you for a couple of weeks?" "Do you have medical insurance to cover office visits and tests?"
	1- Does not ask about any of these.
	2- Asks one such question.
	3- Asks two such questions.
	4- Asks three or more such questions.
Share Information	
9	Explain the status/diagnosis/teach something/patient education/self-care/Counseling using language I understand (avoid medical terminology or otherwise explain it; display info on Electronic Health Record-EHR in helpful manner)
	1- Does not share a diagnosis or information (skips ahead to tests, medications, etc.).
	2- Gives a diagnosis/condition/symptoms/etc. with little explanation.
	3- Gives and explains a diagnosis/condition/symptoms/etc.
	4- Above plus assesses health literacy ("Have you ever heard of ___?" "Do you know anyone with ___?").
10	Elicit concerns and/or questions about illness and treatment (e.g. "This is a lot of information to take in today. What questions do you have for me at this point?")
	1- Does not elicit questions from patient.
	2- Asks once. E.g., "Do you have any questions?"
	3- Incorporates multiple opportunities for patient to ask questions.
	4- Above plus-invites/encourages/legitimizes questions: "What questions do you have?" "We sure have covered a lot today; what questions do you have at this point."
Reach Agreement on Problems and Plans	
11	Involve patient in decisions as much as he/she wants and is appropriate to the context. (e.g. offer "options" and get patient's "preferences")
	1- Lays out the plan.
	2- Lays out the plan and checks for understanding and/or questions.
	3- Gets Patient input into the plan, e.g. preferences for therapy/drugs/surgery and other options.
	4- Asks patient to suggest a plan (when appropriate). Note, this is especially important when discussing lifestyle changes (diet, exercise, smoking, weight, activity, etc.)
12	Promote Patient Empowerment by praising efforts to maintain and/or improve health. E.g. Praise or complement any of the following: maintaining a health lifestyle, being in overall good health, maintaining or improving healthy habits (caffeine/alcohol /tobacco/diet/exercise-any one or combination),managing a chronic illness, reframing failed attempts to improve habits as an important step and something to build upon, presentation to healthcare arena at this appointment when something is worrying.
	1- No acknowledgement of patient's efforts to maintain/improve a healthy lifestyle.
	2- Some acknowledgement of patient's effort to maintain/improve a healthy lifestyle.
	3- Good acknowledgement of patient's efforts to maintain/improve a healthy lifestyle.
	4- Strong and repeated acknowledgement of patient's effort to maintain/improve a healthy lifestyle.
Provide Closure	
13	Provide content closure (brief summary, indication of next steps, discuss follow-up).
	1- No content closure or ran out of time.
	2- 1 element done.
	3- 2 elements done.
	4- All 3 elements done plus asked if there are any other additional questions or concerns.
14	Provide personal closure (thank them, eye contact/smile, and personal goodbye). Examples of "personal goodbye": 'it was nice meeting you', 'I hope this will help you.' 'See you next time.'
	1- No personal closure or ran out of time.
	2- 1 element done.
	3- 2 elements done.
	4- All 3 elements done with warmth and sincerity.
	Comments:
	WVSOM copyright 2006: Swarm, Lovett, Nazar. Revised 6/24/21.
